# Impacts, Learner Diversity, and Curricular Framework of a Virtual Global Health Elective Catalyzed by the COVID-19 Pandemic

**DOI:** 10.5334/aogh.4060

**Published:** 2023-05-23

**Authors:** Maria Alonso Luaces, Michelle S. Cochran, Sarah Finocchario-Kessler, Kimberly Connelly, Barbara Polivka, Robin Young, Geoffrey Anguyo, Charles Nwobu, Jessica Evert

**Affiliations:** 1 Department of Family Medicine and Community Health, Director-Office of Diversity and Inclusion, University of Kansas School of Medicine, Kansas City, KS, US; 2 Clinical Assistant Professor, University of Kansas School of Nursing, Kansas City, KS, USA; 3 Department of Family Medicine & Community Health, University of Kansas Medical Center, Kansas City, KS, USA; 4 KUMC Senior International Officer, Director of the Office of International Programs, the University of Kansas Medical Center, Kansas City, KS USA; 5 School of Nursing, the University of Kansas Medical Center, Kansas City, KS USA; 6 Executive Director, Child Family Health International, US; 7 Founder, Kigezi Healthcare Foundation (KIHEFO) Uganda, UG; 8 Director ICATCH AAP & Global Health projects, Princess Marie Louise Children’s Hospital, Accra and Medical Director for Ghana, Child Family Health International, US; 9 Assistant Clinical Professor, UCSF Department of Family and Community Medicine, Global Medical Director, Child Family Health International, US

**Keywords:** global health education, competency-based education, virtual education, health professions education, planetary health, diversity, inclusion

## Abstract

**Background:**

Place-based international electives that build global health competencies have existed for decades. However, these electives require travel and are infeasible for many trainees around the world, particularly those with insufficient financial resources, logistical complexities, or visa limitations. The emergence of virtual approaches to global health electives, catalyzed by the travel pause related to the COVID-19 pandemic, necessitates the exploration of learner impacts, participant diversity, and curricular frameworks. Child Family Health International (CFHI), a non-profit global health education organization that partners with universities to expand immersive educational offerings, launched a virtual global health elective in 2021. The elective drew on faculty from Bolivia, Ecuador, Ghana, Mexico, the Philippines, Uganda, and the United States.

**Objective:**

This study aimed to describe a newly developed virtual global health elective curriculum and evaluate the demographics of and impacts on trainee participants.

**Methods:**

Eighty-two trainees who were enrolled in the virtual global health elective from January to May 2021 completed both 1) pre- and post-elective self-assessments of domains of competency mapped to the elective curriculum and 2) free text responses to standardized questions. Data were analyzed through descriptive statistical analysis, paired t-testing, and qualitative thematic analysis.

**Findings:**

The virtual global health elective had 40% of its participants hail from countries other than the United States. Self-reported competency in global health broadly, planetary health, low resource clinical reasoning, and overall composite competency significantly increased. Qualitative analysis revealed learner development in health systems, social determinants of health, critical thinking, planetary health, cultural humility, and professional practice.

**Conclusion:**

Virtual global health electives effectively develop key competencies in global health. This virtual elective had a 40-fold increase in the proportion of trainees from outside the United States, compared to pre-pandemic place-based electives. The virtual platform facilitates accessibility for learners from a variety of health professions and a wide range of geographic and socioeconomic environments. Further research is needed to confirm and expand on self-reported data, and to pursue approaches to greater diversity, equity, and inclusion in virtual frameworks.

## Introduction

As the field of global health has been defined and matured over the past two decades, so has the practice of global health education [1,2,3,4,5]. Prior to the pandemic, experiential global health education had grown exponentially over the preceding years as student interest and institutional programs had waxed [6,7,8]. The COVID-19 pandemic impacted all sectors of society, including global health education, health professions training, and higher education [9,10,11]. In high income countries (HIC), disruptions in travel created concerns about the sustainability of global health education, which at times myopically focuses on locations abroad. However, the pandemic impacts were disproportionately felt in already resource-constrained environments within low and middle-income countries (LMIC) [12,13,14,15,16,17,18]. Global health education programs have made many pivots, including transitioning experiential learning from in-person to virtual frameworks.

While institutions pivoted to provide education virtually rather than not at all, challenges and losses accompanying the shift from in-person education, both classroom-based and experiential, include deprivation of face-to-face interactions, group discussions, discomfort with interculturality related to sensitive topics, and connectivity/technology limitations [19,20,21,22,23]. It has been noted that the pause necessitated by the COVID-19 pandemic provided an opportunity to re-examine best practices, ethical integrity, inclusion, and many other ideals of global health education programs [24].

Global health experiences aim to advance the competencies of health professions trainees in various ways, including health systems comparisons, interculturality, self-awareness, social justice orientation, commitment to primary care or underserved practice, and awareness of social determinants of health [25,26,27]. Despite the growth in the number of trainees pursuing place-based global health opportunities (onsite learning in a different country), increasing the participation of students from minoritized backgrounds persists as one of the biggest challenges in international education. Students accessing global health education opportunities are a very homogenous group, with approximately 70% of US students who studied abroad in 2019 identifying as white. Additionally, destinations of choice by the predominately white US students overwhelmingly favor European countries, with 40% of students studying in just five countries: the United Kingdom, Italy, Spain, France, and Germany [28].

Growing awareness of the impacts of colonialism, power asymmetries, the perception of an internship abroad as an elite experience, and, more recently, the COVID-19 pandemic have accentuated the need for higher education institutions to rethink global health curricula, access, and inclusivity [24,29,30]. Global health educational programming is both disproportionately accessible to and created by individuals and institutions in HIC [30,31]. Consequently, virtual experiential education is emerging as an innovative, cost-effective pedagogical strategy for achieving global health educational competencies while facilitating greater access for learners from all socio-economic environments. A virtual, almost placeless, environment holds promise as a third space that “both suspends the hierarchical frameworks historically imposed by formal institutions and establishes new frameworks for shared learning that draws on the motives and experiences of all participants.” [32] The virtual format allows for a multiplicity of countries and voices, which are not bounded by geographical location, to engage in a learning environment that might promote more balanced partnerships and assist with the decolonization of knowledge legitimacy and production in global health education student encounters [33]. Virtual border crossing could shift from studying or visiting “the other” to engaging as a group in meaningful cross-cultural encounters where listening, sharing, and multidirectional power neutral exchanges thrive. Finally, for a fraction of the cost, students from a wide range of abilities, economic, social and geographical backgrounds, can participate [34].

### Framework Overview: Virtual Global Health Elective | Child Family Health International (cfhi.org)

This paper describes the virtual global health elective developed by Child Family Health International (CFHI), a United Nations-recognized non-profit organization, headquartered in the United States, with a global team representing 11 countries. Students from over 40 countries have participated in CFHI global health education programs in the organization’s 32-year history. Prior to the launch of the completely virtual portfolio during the pandemic travel suspension, almost all (99%) learners who were enrolled in global health programs through CFHI (students/trainees/professional learners) were from HICs. We evaluated the impacts of the virtual global health elective on learner development and described lessons learned that could improve the accessibility of global health education for diverse students and trainees.

CFHI’s virtual global health electives are designed for final year medical students, advance practice nursing students, and final year allied health students, as well as entry-level professionals, including medical residents, nurse practitioners, and clinical officers. Trainees can either apply directly to the elective or universities/schools can enroll cohorts of students or individual students. Educational opportunities are available in 10 countries (Argentina, Bolivia, Costa Rica, Ecuador, Ghana, India, Mexico, Philippines, South Africa, and Uganda). The educational model utilizes asset-based community development and Fair-Trade Learning frameworks with community-based faculty leaders in the local healthcare and social service systems [35]. Organizational strategic planning, curricular design, and standardized practices across sites are approached collaboratively by the global team, central leadership team, and board of directors.

The virtual elective was created in response to the suspension of place-based, in-person electives. Faculty for the virtual elective drew on the global CFHI team and invited faculty. Invited faculty were from Albert Einstein College of Medicine, Consortium of Universities for Global Health (CUGH), St. Luke’s Medical Center College of Medicine William H. Quasha Memorial and the Planetary Health Alliance. The elective had synchronous and asynchronous components over 4 weeks (40 hours per week). Students were encouraged to attend live synchronous sessions, but sessions were recorded for asynchronous viewing. Zoom and Google Classroom were used to facilitate virtual interactions and manage learning. A pass/fail grading system was utilized based on the evaluation of learners’ e-portfolios that included collated assignments from each section and intercultural personal development plans. The elective curriculum included nine sections: Biomedical and Global Health Ethics, Low Resource Clinical Reasoning, Cross-Cultural Effectiveness and Adaptability, Planetary Health, Health Systems Comparatives, Foundations of Global Health, Community-Based Immersion, Local-Global Health, and Critical Reflection. The global team members facilitated the community-based immersion in Uganda and Ghana. Two intercultural effectiveness and adaptability tools were utilized—the Global Engagement Survey (GES), provided through a partnership with Aperian Global, and the Intercultural Effectiveness Scale, through a partnership with the Kozai Group. The virtual elective tuition for students from HIC was $495, with full tuition scholarships offered to trainees from low and middle-income countries (LMIC).

## Methods

This evaluation used a pre-post self-assessment design. The following students were eligible to apply and participate in the virtual elective: 4th year medical students as well as interdisciplinary health professions students (medical, PA, NP, allied health, residents, and fellows). Ninety students enrolled in the virtual global health elective between January and May 2021. Of these, 82 students completed a 7-item pre-elective self-assessment, and 68 students completed the parallel 7-item post-elective self-assessment immediately after the conclusion of the elective, with 52-students completing both the pre-and post-elective self-assessment.

Students rated their knowledge, skills, and attitudes toward global health and their competencies related to low resource clinical reasoning, with response options ranging from 1 = None to 5 = Excellent. Students were also asked if they understood and could articulate concepts of planetary health, global health, and global health ethics and bioethics, with response options from 1 = Strongly Disagree to 5 = Strongly Agree. Lastly, students were asked if they understand and practice cross-cultural effectiveness and adaptability and if they have an appreciation of health systems comparatives from around the world, with response options from 1 = Strongly Disagree to 5 = Strongly Agree. The post elective self-assessment also included one open-ended question to assess any personal or professional impact of participation in the virtual elective.

The surveys were de-identified using a serial numeric code. The numeric code is linked to student identifying information, but the document linking the student information to the numeric code was maintained securely by CFHI. Only surveys with de-identified numeric codes were shared with investigators at the University of Kansas Medical Center (KUMC) for analysis. The Institutional Review Board (IRB) at the University of Kansas Medical Center determined the project was non-human subjects research, thus it did not require IRB approval.

### Data analysis

Demographic data were analyzed descriptively. The scores for the 7-item self-assessment measures were summed for total pre- and post-scores. Paired t-tests were performed for the 52 students with pre- and post-data available. Significance was set a priori at an alpha of 0.05.

Thematic analysis was used to code comments and establish themes from one open-ended survey question: “In your own words, how did this elective impact you as a person, professional, and any other ways?” The written responses were inductively coded through an iterative process of coding, comparing, consolidating and refining by two researchers. The two researchers consulted with each other often as they worked on generating initial codes using the global health competencies as a coding framework. Codes were then discussed with two additional members of the research team and collectively categorized items while extracting vivid, compelling examples from the data.

## Results

Students (N = 90) who participated in virtual global health electives through CFHI were primarily female (60%), and either White (38.9%), Hispanic/Latino (20.1%), Black/African American (15.6%), or Asian (15.6%). Of the total students, 60% were from North America, 17.8% were from Central/South America, 10% were from Africa, and 8.9% were from the Middle East. Among the whole, 68% of the learners were medical students, 8.9% were physician’s assistant students, and 6.7% were public health students (Table 1).

**Table 1:** Participant demographics (N = 90).


CHARACTERISTIC	N (%)

Sex	

Female	54 (60)

Male	36 (40)

Ethnicity	

African	2 (2.2)

Asian	14 (15.6)

Black/African American	14 (15.6)

Hispanic/Latino	19 (20.1)

Middle Eastern	5 (5.6)

White	35 (38.9)

Biracial	1 (1.1)

Region/Country of Residence & University North America (United States)	54 (60)

Boston University School of Medicine Clemson University	

Medical University of South Carolina Rutgers University	

Touro University, California	

University of Arizona College of Medicine University of Kansas School of Medicine University of Kentucky College of Medicine University of Michigan Medical School Wayne State University School of Medicine	

Pacific Northwest University of Health Sciences College of Osteopathic Medicine	

Africa (Egypt, Kenya, Somalia, Uganda) Addis Ababa Medical University College Alexandria University, Faculty of Medicine Kabale Institute of Health Sciences	9 (10)

Kampala Institute of Science and Technology Masinde Muliro University of Science and Technology University of Nairobi	

Centra/South America (Costa Rica, Peru)	16 (17.8)

Universidad de Costa Rica	

Universidad Peruana de Ciencias Aplicadas	

Asia (Pakistan, Bhutan)	2 (2.2)

Al Nafees Medical College, Isra University	

Middle East (Israel, Lebanon) American University of Beirut Lebanese American University	8 (8.9)

Rose-Marie and Chagoury	

Lebanese American School of Medicine	

Technion American Medical School	

Europe (England)	1 (1.1)

Cardiff University	

Level of trainee	

Medical student (1st/2nd year)	21 (23.3)

Medical student (3rd/4th yr./senior level)	40 (44.4)

Physician Assistant student	8 (8.9)

Nursing student (undergraduate/graduate)	4 (4.4)

Allied Health/Other Health Profession	1 (1.1)

Public Health student (undergrad/graduate)	6 (6.7)

Resident physician	3 (3.3)

Post-baccalaureate student	2 (2.2)

Undergraduate, Pre-Medical/Pre-health	3 (3.3)

Not a student (Pediatrician; Gap year)	2 (2.2)


Comparisons of mean scores per item for each trainee for whom pre and post self-assessment data could be paired (N = 52) revealed that learners significantly increased their scores (improved) for 5 of the 7 items (Table 2). There was also a significant increase in total scores, indicating an overall improvement in knowledge and attitudes.

**Table 2:** Paired t-test per each item, and summed score. (N = 52).


ASSESSMENT ITEM	PREM (S.D.)	POST M (S.D.)	P

1. Compared to my professional/trainee peers, my current knowledge, skills, and attitudes related to global health.^a^	3.2 (.8)	4.1 (.7)	<.001

2. I understand and can articulate the concept of planetary health and provide examples of planetary health.^b^	2.7 (1.0)	4.6 (.6)	<.001

3. I understand and can articulate the concept of global health and provide examples of global health. ^b^	3.3 (.9)	3.9 (1.6)	.019

4. I understand and can articulate concepts of global health ethics and bioethics. ^b^	3.2 (.9)	3.7 (1.6)	.042

5. My competency (knowledge, skills, attitudes) related to low resource clinical reasoning. ^a^	3.0 (.8)	4.0 (.7)	<.001

6. I understand and practice cross-cultural effectiveness and adaptability. ^b^	3.6 (.9)	3.9 (1.4)	.079

7. I have an appreciation of health systems comparatives from around the world. ^b^	3.6 (.8)	4.0 (1.3)	.079

Total Score (scores can range from 7–49)	22.7 (3.6)	28.4 (5.0)	<.001


*Note*: ^a^ Response options: 1 = None, 2 = Below Average, 3-Average, 4 = Above Average, 5 = Excellent. ^b^ Response options 1 = Strongly Disagree, 2 = Disagree, 3 = Neutral, 4 = Agree, 5 = Strongly Agree.

A total of 68 trainees responded to the open-ended question. The following themes were identified: Increased awareness of global health systems, deeper understanding of social determinants of health, critical thinking around global health ethics, awareness of planetary health, informed future clinical practice, and cultural humility. Table 3 includes the identified themes and an excerpt of statements from learners.

**Table 3:** Personal and professional benefits of participation in the virtual global health elective.


THEMES	EXEMPLARY QUOTES

Increased awareness of global health and global health systems	“This elective really opened my eyes on how expansive the field of global health is, what the challenges that one might face are and how to effectively tackle them.” “I now understand more in depth the different factors that affect the global healthcare infrastructure.”

A deeper understanding of the interactions between biological and social determinants of health.	“I developed understanding of the interplays between disease processes, poverty, social circumstances, culture, geopolitical realities, historical contexts, and the complexities of health and wellness.”

Developed critical thinking around global health ethics	“It opened my eyes to ethical issues, and key issues within global health that I was previously blind to.” “Made us really reconsider ethics when it comes to providing medical help/treatment in other countries.”

Significant increase in awareness of planetary health	“This elective educated me and made me realize the importance of planetary health and my role as a future physician in taking care of the planet in addition to human beings, because of the effect that the environment has on us.”

Informed future professional practice	“Learned about low resource clinical reasoning.” “This elective impacted me as a person, teaching me new skills to put in practice in my country, regarding global health.”

Developing cultural humility and deeper human connections through respectful interactions.	“This elective made me think more about how I can actively seek to understand cultures different than my own and to also think about others’ perspectives when I am communicating with people from cultures different than my own.” “It really helped me develop my skills and make new international friends.”


Trainees in the virtual global health elective reported both professional and personal impacts. Learners described enhanced knowledge and awareness of global health and global health systems. Additionally, many comments revealed learners’ understanding the impact of the social determinants of health on the wellbeing of individuals within a community and a significantly heightened awareness of the connection between planetary health and human health. At the interpersonal level, learners commented on how the interactions with diverse learners in their virtual elective classroom allowed for increased appreciation for cultural perspectives and improved communication due to this increased cultural awareness.

## Discussion

This study provides insights into learner development and the diversity of participants when a place-based global health elective is adapted to a virtual context, as necessitated by the COVID-19 pandemic. The impacts on learner development were in both personal and professional realms, and on the organization’s ability to provide greater access to the training. The curriculum maintained a community immersion component while utilizing didactic discussion, presentation, and case-based approaches. In terms of learner development, there was a statistically significant improvement in the domains of competency within general global health, planetary health, and low resource clinical reasoning, as well as the composite score for all domains. Qualitative data further demonstrated increased awareness of the relationship between human health and environmental/animal health, social determinants of health, and cultural humility/intercultural effectiveness. These findings suggest that virtual electives successfully nurture global health competencies and professional development. The furthered competencies also align with undergraduate and graduate medical education competency targets, including interpersonal and communication skills, professionalism, and systems-based practice [8, 36].

Program accessibility to LMIC learners and professions was realized through the elective, with 38% of elective students being from LMIC. Of those, 100% received full scholarships. In comparison, for CFHI’s place-based programs in 2019, 4 out of 883 (<1%) trainees were from LMIC. While the trainees participating in the virtual elective were more diverse than those in the traditional version, and scholarships helped enable this inclusion, it is recognized that there were still aspects of the elective that were not optimally inclusive. For example, the instructional language of the elective was English, the time of synchronous content was based on daytime hours in the four United States time zones, and the elective did not provide accessibility for hearing or visually impaired trainees. Despite these shortcomings, the improvement in inclusion and diversity of trainees because of available scholarships and the virtual framework is remarkable. The reasons for this are multiple, including lower cost (the virtual elective is $495, compared to $2500 plus airfare and spending money), there being no visa attainment nor travel needed, the ease of scheduling this experience alongside other academic pursuits, and the ability to maintain other commitments at home (parenting, jobs, school, etc.).

Virtual exchange in non-patient care contexts is a less ethically precarious platform for global health clinical education than place-based education in terms of the ability to ensure safe patient care and adherence to global health ethics and principles. When learners and faculty are physically in low-resource international settings, there is a high risk for ethical and patient safety missteps [29,37]. Further, documented extensively in the literature relative to place-based global health experiences, is the danger of power differentials that allow inexperienced trainees to provide care to marginalized patients, thus lowering the standards of care below what would be required in the United States [38,39,40]. Virtual platforms reduce this risk. The virtual elective also prohibited any video recording of patient care interactions, with only case-based approaches utilized. Class activities also included mock patient interactions based on actual scenarios in select programs to avoid voyeurism and sub-optimal ethical integrity, which would undoubtedly still be an issue if virtual programs allowed trainees to be virtually involved in live patient care. Given that virtual health electives are in their infancy, virtual patient encounters should be avoided, since without proper planning and partnerships, the benefits would likely only be for the trainees leveraging their privilege to gain access to patient care in patient populations who often cannot say “no” when asked to provide permission. Importantly, this evaluation demonstrates significant impacts to learner development in key domains without live clinical content.

There are several weaknesses of this study, including self-reported data, lack of review of students’ e-portfolios for evaluation purposes, and drawing on one organization’s experience over a five-month period. The survey questions were not directly linked to demonstration of global health competencies, limiting conclusions on improved mastery. Although professional development was not directly assessed, this theme emerged from the qualitative data and many of the quantitative measures and qualitative themes had implications for both personal and professional development. Traditional academic institutions will likely face additional hurdles to enroll external trainees in virtual global health electives. Future studies and frameworks should include more rigorous assessment approaches, the consideration of participatory versus acquired competencies, and other aspects of programming necessary to allow for assessment.

In conclusion, this evaluation provides evidence that virtual approaches to global health electives are impactful for learner development and greater diversity of participants. The framework of the elective provides insights for the broader global health education community of practice. Virtual environments can potentially change the face of global health education, including those for whom place-based, in-person experiences have been out of reach while easing the power asymmetries that have historically plagued the field of global health education. Finally, further evaluation should focus on the impacts on faculty and local leadership in partnering communities, curricular delivery approaches, and non-self-report methodology for assessing impact.
